# Comparison of early postoperative dry eye after KLEx and FS-LASIK: a contralateral eye study

**DOI:** 10.1186/s12886-026-04902-w

**Published:** 2026-05-12

**Authors:** Ali Küçüködük, Mustafa Aksoy

**Affiliations:** 1 Department of Ophthalmology, Faculty of Medicine, Karamanoglu Mehmetbey University, Karaman, 70100 Türkiye; 2 Dunyagoz Hospital, Izmir, Türkiye; 3 Konak Vocational School, Opticianry, Izmir Türkiye

**Keywords:** Dry eye, Tear osmolarity, KLEx, LASIK, Contralateral eye study

## Abstract

**Objective:**

To compare early postoperative dry eye outcomes in contralateral eyes of the same patients undergoing Keratorefractive Lenticule Extraction (KLEx) versus Femtosecond Laser-Assisted Laser In Situ Keratomileusis (FS-LASIK), with tear film osmolarity defined as the primary outcome measure.

**Methods:**

This study was conducted at Dunyagoz Izmır Hospital and involved patients who underwent corneal refractive surgery between June 2021 and December 2024. Patients with pre-existing dry eye disease were excluded from the study. Patients underwent KLEx in one eye and FS-LASIK in the fellow eye. Tear film osmolarity (primary outcome) was measured preoperatively and postoperatively using the TearLab^®^ system. Secondary outcome measures included tear break-up time (tBUT) and subjective symptoms assessed via the Ocular Symptom Questionnaire Scores (OSDI).

**Results:**

A total of 34 eyes of 17 patients (7 females and 10 males) were evaluated. Group 1 (KLEx) included 17 eyes and group 2 (FS-LASIK) included contralateral 17 eyes. The mean postoperative follow-up period was 34.2 days. Mean tear osmolarity was 297.7 ± 8.3 mOsm/L in the group 1 and 315.3 ± 10.2 mOsm/L in the group 2 (*p* < 0.001). Mean tBUT was 10.3 ± 3.1 s for group 1 and 7 ± 2.32 s for group 2 (*p* < 0.001). The mean postoperative OSDI scores were 32.5 for group 1 and 42 for group 2, with a preoperative baseline of 10.2 (both groups; *p* < 0.001).

**Conclusions:**

KLEx demonstrated superior early postoperative ocular surface stability compared to FS-LASIK in contralateral eyes. The procedure resulted in significantly less tear hyperosmolarity and better subjective comfort, suggesting it may be a preferable surgical option for minimizing dry eye induced by corneal refractive surgery.

## Introduction

The International Dry Eye Workshop (DEWS) defines Dry Eye Disease (DED) as “a multifactorial disorder of the tear film and ocular surface that results in symptoms of discomfort, visual disturbance, and tear film instability accompanied by potential damage to the ocular surface. It is associated with increased tear film osmolarity and inflammation of the ocular surface” [1]. Dry eye can arise from insufficient tear production or excessive tear evaporation. Clinical signs of dry eye include reduced aqueous tear secretion, decreased tear volume on the ocular surface, increased tear evaporation rate, and elevated tear osmolarity [2].

Corneal refractive surgery is one of the most frequently performed procedures. The first laser in situ keratomileusis (FS-LASIK) surgery was conducted in 1990, and the first keratorefractive lenticule extraction (KLEx) surgery was performed in 2008 [3]. In recent years, FS-LASIK and KLEx surgeries have gained significant popularity as refractive surgical techniques. This is due to their ability to provide rapid and painless visual rehabilitation, as well as their reputation as safe and effective procedures with numerous advantages over other refractive surgical methods [3, 4]. Recent comparative studies and meta-analyses published in the last decade have further solidified the safety profile of lenticule extraction, highlighting its advantages in biomechanical stability and ocular surface preservation compared to flap-based procedures [5, 6].

Post FS-LASIK dry eye is the most common cause of dry eye following ophthalmic surgeries, with up to 95% of patients reporting some degree of dry eye symptoms immediately after FS-LASIK [7]. The primary reasons for this include flap-induced loss of corneal innervation, reduced corneal reflex, and impaired blink and meibomian gland reflexes, leading to decreased aqueous and lipid tear secretion, as well as reduced mucin expression [8, 9]. Additionally, diminished trophic effects on the corneal epithelium, damage to limbal goblet cells during suction, inflammation, and medication side effects are other contributing factors to dry eye [10].

KLEx surgery is believed to cause less dry eye in the postoperative period compared to FS-LASIK. This was attributed to its lower impact on the corneal nerves. The mechanisms underlying dry eye following KLEx are thought to be similar to those of FS-LASIK, excluding the neurotrophic effects [2].

To our knowledge, there are no studies in the literature on the results obtained when different surgical techniques are applied to different eyes of the same patient. The primary objective of this study was to compare early postoperative dry eye outcomes in contralateral eyes of the same patients undergoing KLEx versus FS-LASIK. Specifically, we aimed to assess objective ocular surface changes using tear osmolarity (primary endpoint) and tear break-up time (tBUT), alongside subjective symptoms evaluated via the Ocular Symptom Questionnaire Scores (OSDI).

## Methods

In this retrospective study, tear osmolarity results as well as the conventional dry eye tests of the patients who applied to Dünyagöz Izmir Hospital and underwent KLEx surgery in one eye and FS-LASIK surgery in the other eye were compared retrospectively in accordance with the Helsinki Declaration protocol. Ethical approval was obtained from the Karamanoğlu Mehmetbey University Scientific Research and Publication Ethics Committee. Informed consent was obtained from all participants. (Trial Number: 165002)

### Patient selection

This study enrolled patients who underwent complete ophthalmological examinations before and after surgery with no evidence of ophthalmological pathology or postoperative complications. Patients with a preoperative and postoperative visual acuity of 20/20, no history of dry eye complaints prior to surgery, a tBUT value over 10 s, and Schirmer test results over 10 mm were included.

Performing different surgical procedures on each eye of the same patient is not a common practice in routine clinical settings. In this study, the reasons for selecting different surgical techniques in the two eyes of the same 17 patients were as follows: FS-LASIK was preferred for the fellow eye with higher astigmatism, FS-LASIK was preferred over KLEx due to the latter’s lack of torsion detection capabilities. In addition, some patients who underwent FS-LASIK could not undergo KLEx due to a lack of myopic spherical value.

Eyes in which KLEx surgery was preferred were designated as Group 1, whereas the other eyes that underwent FS-LASIK surgery were designated as Group 2.

### Surgical procedures

KLEX Procedure for KLEx: The operation was performed using a Visumax 500 femtosecond laser platform (Carl Zeiss Meditec AG, Jena, Germany). A drop of proparacaine hydrochloride 0.5% (Alcaine) was instilled in each eye, followed by preparation of lids and periocular skin with povidone-iodine (betadine). After sterile draping and placement of the lid speculum, KLEX surgery was performed without complications. All the patients had a anterior corneal cap thickness of 120 μm and an optical zone of 6.7 mm. A anterior corneal cap side-cut size of 3 mm was established as the standard. All surgical procedures were performed by the same surgeon. (MA)

FS-LASIK procedure: Flap creation was performed using a Visumax 500 femtosecond laser platform (Carl Zeiss Meditec AG, Jena, Germany). A drop of proparacaine hydrochloride 0.5% (Alcaine) was instilled into each eye, followed by preparation of lids and periocular skin with povidone-iodine (Betadine). After sterile draping, a lid speculum was placed and FS-LASIK flap surgery was performed without complications. All flap diameters were set at 8.8 mm and all flap thicknesses were set at 100 microns. The patients were then transferred to an EX-500 (Alcon Laboratories, Inc., Fort Worth, TX, USA). All patients underwent an uncomplicated standard FS-LASIK procedure with 6.5 mm optical zone ablation at the interface after flap dissection. All surgical procedures were performed by the same surgeon. (MA)

Postoperative treatment consisted of the administration of 0.1% dexamethasone drops four times a day for two weeks and 0.5% moxifloxacin drops four times a day for all patients. Furthermore, 1.5 mg/ml sodium hyaluronate drops were administered six times daily for a period of six months.

### Tear film osmolarity measurement

Ocular osmolarity was measured using an osmometry test developed by TearLab. (The TearLab Osmolarity System is manufactured by TearLab Corporation, a company headquartered in the United States, San Diego, California) The osmometer works as follows: a microelectrode measures the number of charged particles in the tear sample; this electrode was designed to avoid direct contact with the ocular surface, thereby reducing the possibility of reflex tear production. The measurement method appears to be as accurate as more comprehensive osmometers, with an average difference of only 2 mOsm/L between normal and dry eye patients [3]. While 316 mOsm/L threshold was thought to better discriminate between mild and moderate/severe dry eye, 308 mOsm/L is now considered a widely accepted threshold [11].

### Ocular symptom questionnaire scores (OSDI)

To assess subjective symptoms, the OSDI score questionnaire was completed for each subject [12]. The OSDI was introduced in 1997 by the Outcomes Research Group at Allergan Inc. Twelve questions are assessed in three subtopics that evaluate symptoms, functional limitations and environmental factors related to dry eye disease [13]. There are five response options for the first subtopic (Always; Most of the time; Half of the time; Sometimes; Never). For the remaining two subtopics, there is an additional response option (not applicable). The overall OSDI and subtopic scores ranged from 0 to 100. OSDI scores defined the ocular surface as normal (0–12 points) or mild (13–22 points), moderate (23–32 points), or severe (33–100 points) dry eye disease [13,14]. To quantitatively assess subjective ocular surface conditions, Turkish versions of the OSDI were used.

Patients had completed three OSDI questionnaires during the first postoperative month.

The first questionnaire evaluated the eye that underwent KLEX, the second assessed the eye that underwent FS-LASIK, and the third questionnaire was completed to evaluate the preoperative ocular surface condition.

Outcome Measures: The primary outcome measure defined for this study was the postoperative tear film osmolarity. Secondary outcome measures included subjective symptoms assessed via the OSDI score and tear film stability measured by tBUT.

### Statistical analysis

All statistical analyses were performed using SPSS software for Windows (version 22.0; IBM Co., Chicago, IL, USA). Statistical analyses of the OSDI questionnaire scores for eyes that underwent FS-LASIK and KLEx surgery, as well as comparisons of tear osmolarity measurements between the two groups, were conducted using the Mann–Whitney U test. In addition, the preoperative and postoperative OSDI questionnaire data were analyzed using the Paired Sample T test. A p-value < 0.05 was considered statistically significant.

All data are presented as mean ± standard deviation (mean ± SD).

## Results

A total of 34 eyes from 17 patients were included in the study. KLEx was performed in the right eye and FS-LASIK in the left eye in 11 patients, while in 6 patients, the procedures were reversed. This distribution showed a statistically significant difference (*p* < 0.001). The mean age of the patients was 25.3 years. Seven of the patients were female and ten were male. Measurements were taken in 17 eyes of patients who underwent KLEx surgery in group 1 and in the other 17 eyes of patients who underwent FS-LASIK surgery in group 2. The mean postoperative time was 34.2 days. The refractive error data for Groups 1 and 2 are given in Table 1.

**Table 1:** Baseline refractive characteristics of the study groups

	Group 1	Group 2	*p*
Number of eyes	17	17	
Sphere (D)	-4.2±1.1	-1.24±0.21	**< 0.001**
Cylinder (D)	-0.65±0.09	-2.96±0.98	**< 0.001**
Spherical equivalent	-4.53 ± 1.10 D	-2.72 ± 0.53 D	**< 0.001**

D: Dioptre

### Tear Breakup Time (tBUT)

Preoperative and postoperative tBUT values and changes for both groups are given in Table 2. A significant difference was observed between the two groups with regard to the postoperative decrease in tBUT (*p* < 0.001). Furthermore, the postoperative decrease in tBUT values within the groups were found to be statistically significant in both groups (*p* < 0.001). Mean tBUT values were 10.3 ± 3.1 s for group 1 and 7 ± 2.32 s for group 2. There was a significant difference between the two groups (*p* < 0.001).

**Table 2:** Comparison of preoperative and postoperative tBUT values between the groups

	Group 1 (sec)	Group 2 (sec)	*p*
Preoperative	14.2 ± 3.21	14 ± 3.19	0.06
Postoperative	10.3 ± 3.1	7 ± 2.32	**< 0.001**
p	**< 0.001**	**< 0.001**	

Sec: Second

### Tear film osmolarity measurement

**Fig. 1:**
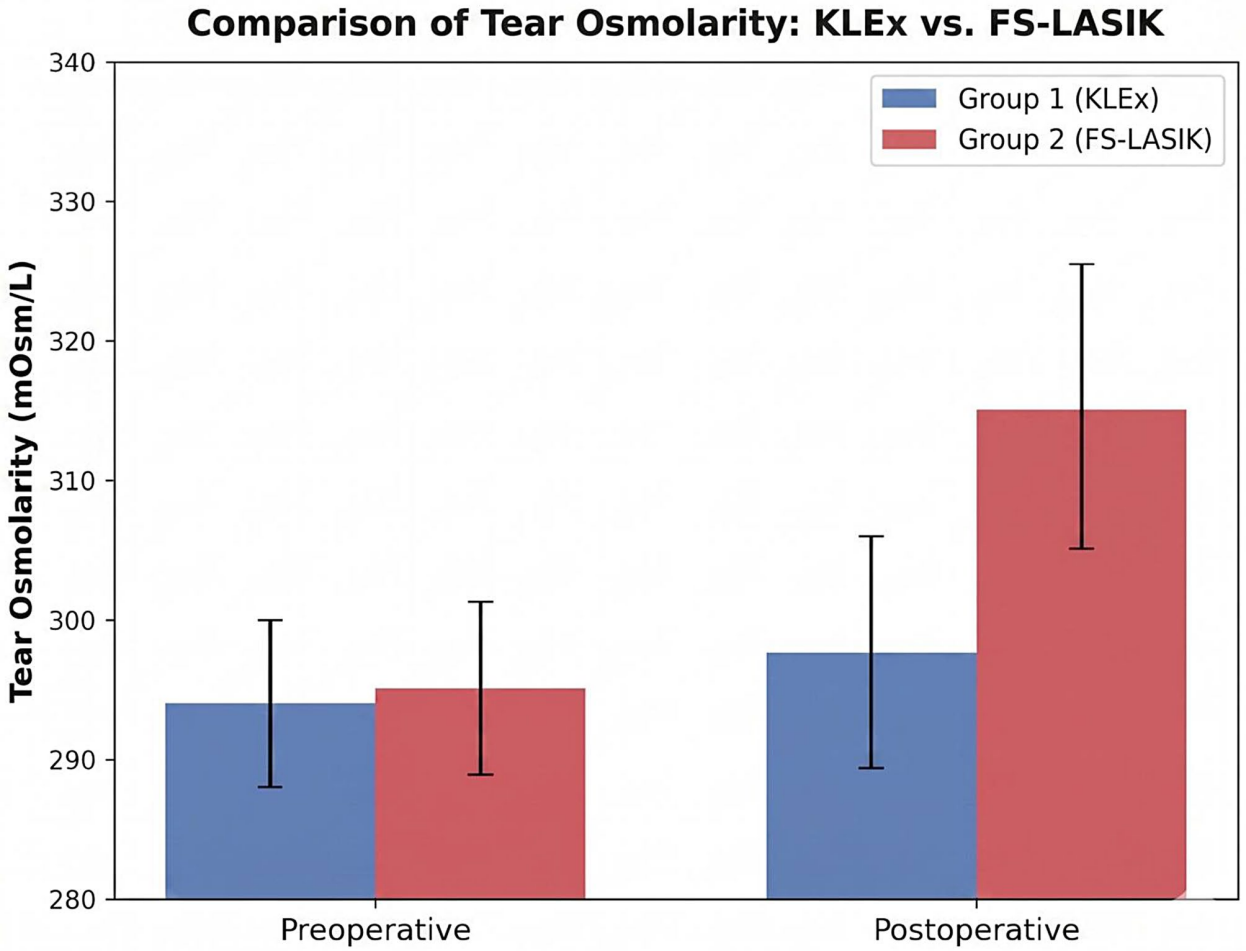
Comparison of preoperative and postoperative mean tear osmolarity levels between Group 1 (KLEx) and Group 2 (FS-LASIK). Note the significant increase in osmolarity in the FS-LASIK group postoperatively compared to the relative stability in the KLEx group. Error bars represent standard deviation. (*p* < 0.001 for postoperative comparison)

Preoperative and postoperative mean tear osmolarity values and changes for both groups are given in Table 3. A significant difference was observed between the two groups with regard to the postoperative increase in tear osmolarity (*p* < 0.001). Moreover, the postoperative increase in tear osmolarity values was also found to be statistically significant within the groups (both groups, *p* < 0.001). Mean osmolarity values for group 1 were 297.7 ± 8.3 mOsm/L (290–306 mOsm/L) and 315.3 ± 10.2 mOsm/L (298–331 mOsm/L) for group 2. There was a significant difference between the two groups (*p* < 0.001). Preoperative and postoperative tear osmolarity measurements for both groups are illustrated in Fig. 1.

**Table 3:** Comparison of preoperative and postoperative tear film osmolarity between the groups

	Group 1 (mOsm/l)	Group 2 (mOsm/l)	*p*
Preoperative	293 ± 6.9	292.2 ± 7.1	0.09
Postoperative	297.7 ± 8.3	315.3 ± 10.2	**< 0.001**
p	**< 0.001**	**< 0.001**	

### Ocular symptom questionnaire scores

According to the results of the OSDI score questionnaire, there was a significant difference between the preoperative score (mean, 10.2) and the postoperative score between group 1(32.5 mean) and group 2 (42 mean). (*p* < 0.001) There was also a significant difference between group 1 and group 2 eyes in the postoperative period and this score was in favor of group 1. (*p* < 0.001) All patients reported that their preoperative OSDI evaluations were similar for both eyes, indicating comparable baseline ocular surface status. In addition, a statistically significant difference was observed between the preoperative and postoperative OSDI scores in both eyes. (*p* < 0.001)

## Discussion

This study demonstrates that eyes who underwent KLEx had significantly lower tear osmolarity levels in the early postoperative period compared to those who underwent FS-LASIK. In addition, tBUT levels were significantly higher in patients who underwent KLEx and OSDI scores were measured lower in the KLEx group. These findings suggest that tear film stability is achieved more easily in patients who underwent KLEx in the early postoperative period and that dry eye symptoms may be less frequent. While our findings align with existing literature on the impact of refractive surgery on the ocular surface, this research uniquely employs a within-patient comparison of different methods, eliminating inter-subject variability.

OSDI scores and tear osmolarity are considered to be the most useful instruments for the assessment of dry eye severity and postoperative patient satisfaction [15, 16]. OSDI scores quantitatively measure subjective symptoms reported by the patient. A high OSDI score is directly proportional to dry eye severity and can be administered repeatedly during the postoperative period to monitor changes in patient comfort. Tear osmolarity, on the other hand, measures the charged particles in the tear sample. High levels indicate tear film instability and thus serve as an objective marker of dry eye. Sullivan et al. stated that tear osmolarity is an important biomarker for dry eye disease and postoperative ocular surface stability. In their study, they compared conventional tests on patients with dry eye syndrome and showed that tear osmolarity was the strong predictor of disease severity in the normal, mild/moderate, and severe categories [17]. In addition, Lee et al. reported that conventional dry eye tests (Schirmer, BUT) were not correlated with corneal nerve regeneration after refractive surgery [18]. They further suggested a possible relationship between tear osmolarity and corneal nerve regeneration; however, this observation should be interpreted with caution and warrants further investigation. All types of corneal refractive surgery are known to frequently cause dry eye symptoms. The majority of studies in the literature support the hypothesis that the most important factor in the pathophysiology of dry eye and decreased corneal sensitivity due to refractive surgery is the interruption of corneal nerves in the anterior third of the corneal stroma during surgery [19]. More recent investigations utilizing in vivo confocal microscopy have corroborated these findings, demonstrating significantly faster recovery of subbasal nerve plexus density and corneal sensation in eyes undergoing lenticule extraction compared to FS-LASIK [20,21]. Corneal nerve transection during refractive surgery may subsequently lead to suppression of tear secretion from the lacrimal gland, decreased mucus expression in the corneal epithelium, and frequent blinking, as these homeostasis-maintaining behaviors are driven by a neuronal feedback loop mediated by corneal sensitivity [22,23]. However, the majority of studies in the current literature on dry eye after KLEx support that KLEx has less adverse effects on ocular surface parameters, provides faster improvement in corneal sensitivity, and provides better short- and long-term results compared to FS-LASIK [24]. In our study, although the degree of myopia was higher in the KLEx group, the total stromal lenticule volume removed was also greater than the total stromal ablation in the FS-LASIK group. Nevertheless, the KLEx group demonstrated better ocular surface stability. This finding suggests that preservation of the subbasal nerve plexus, facilitated by the small incision approach in KLEx, may represent a more dominant factor in preventing postoperative dry eye than the overall volume of corneal tissue removal.

It is stated that damage to the corneal nerves during FS-LASIK flap formation affects the lacrimal functional unit by causing instability and high osmolarity levels in the tear film [2, 25]. Wong [26] et al. reported that KLEx results in less corneal nerve transection compared to FS-LASIK due to the smaller corneal incision. This advantage resulted in a reduced incidence of dry eye symptoms and a more rapid improvement in tear film parameters. It is noteworthy that the FS-LASIK group in our study had significantly higher preoperative astigmatism compared to the KLEx group (-2.96±0.98 D vs. -0.65±0.09D), necessitating different ablation profiles. However, current literature suggests that the primary driver of refractive surgery-induced dry eye is the severance of corneal nerves during flap creation rather than the stromal ablation depth itself. Furthermore, the decrease in corneal subbasal nerve fiber density was reported to be more severe in FS-LASIK patients than in KLEx patients in the early postoperative period [27]. Furthermore, existing literature suggests that increased tear film osmolarity and reduced tBUT values not only influence the severity of dry eye symptoms but may also contribute to postoperative refractive errors. This effect is thought to be related to alterations in corneal surface regularity [28, 29].

Several studies have demonstrated that inflammatory mediators play a crucial role in ocular surface changes after surgery. Gao et al. reported that levels of interleukin-6 (IL-6) and nerve growth factor (NGF) were significantly elevated in the early postoperative period following both FS-LASIK and KLEx procedures [26]. However, these levels recovered more rapidly in patients who underwent KLEx. Previous molecular studies have demonstrated that specific inflammatory and wound-healing–related biomarkers are differentially expressed following refractive surgery. In particular, proteins such as Lysozyme C (LYZ), Macrophage Migration Inhibitory Factor (MIF), and Peroxiredoxin-1 (PRDX1) have been shown to exhibit a lower inflammatory response profile in KLEx compared to FS-LASIK [29]. Moreover, transcriptomic and proteomic analyses of tear samples have revealed that key inflammatory mediators, including interleukin-15 (IL-15) and vascular endothelial growth factor A (VEGFA), are significantly more elevated in FS-LASIK than in KLEx during the early postoperative period.30 These biomarkers are associated with inflammatory activation, epithelial healing, and angiogenic signaling, and their lower expression levels in KLEx suggest a reduced inflammatory burden [30, 31]. The findings outlined in the extant literature suggest the importance of maintaining lower osmolarity levels in order to optimize refractive outcomes and patient satisfaction [32].

Although our findings were compatible with the data in the literature, our study had several limitations. Firstly, our follow-up period was limited to approximately five weeks; further studies with longer follow-up are needed to determine whether these differences persist over time. Consequently, the findings reported herein are specifically reflective of the immediate postoperative recovery phase and may not be extrapolated to long-term ocular surface stability without further longitudinal data. Another limitation of the present study was the presence of a statistically significant difference in spherical and cylindrical refractive values between the two eyes, which may have influenced inter-eye comparisons. The other limitation was the relatively small sample size. However, the strength of this study lies in its contralateral eye design, which minimizes confounding variables by using the fellow eye as a control. This design allowed for the detection of statistically significant differences despite the limited cohort size. Future studies should include extended follow-up periods and larger sample sizes to validate these findings. Additionally, investigating the relationship between corneal nerve density, tear film parameters, and osmolarity changes over time may provide further insight into the mechanisms underlying dry eye after refractive surgery.

This study suggests that KLEx may offer advantages over FS-LASIK in terms of early postoperative tear film stability, as demonstrated by lower tear osmolarity values ​​and conventional dry eye tests. To the best of our knowledge, this is the first study to evaluate tear osmolarity and conventional dry eye parameters using a contralateral eye design to compare different surgical techniques. Studies with larger sample sizes, longer follow-up periods, and comprehensive ocular surface assessments are needed to fully understand the long-term implications of these findings.

## Data Availability

The datasets supporting the conclusions of this article are included within the article (and its additional files).

## Abbreviations

KLEx: Keratorefractive Lenticule Extraction
FS-LASIK: Femtosecond Laser-Assisted Laser In Situ Keratomileusis
OSDI: Ocular Symptom Questionnaire Scores
tBUT: Tear Break-Up Time
